# Association of Modic change types and their short tau inversion recovery signals with clinical characteristics- a cross sectional study of chronic low back pain patients in the AIM-study

**DOI:** 10.1186/s12891-020-03381-4

**Published:** 2020-06-10

**Authors:** Lars Christian Haugli Bråten, Elina Iordanova Schistad, Ansgar Espeland, Per Martin Kristoffersen, Anne Julsrud Haugen, Gunn Hege Marchand, Nils Vetti, Are Hugo Pripp, Thomas Istvan Kadar, Jan Sture Skouen, Margreth Grotle, Lars Grøvle, John-Anker Zwart, Jens Ivar Brox, Kjersti Storheim, The AIM-study group, Audny Anke, Maja Wilhelmsen, Terese Fors, Guro Kjos, Ida Beate Østhus, Britt Elin Lurud, Fredrik Granvigen, Hege Andersen, Øystein Petter Nygaard, Vidar Rao, Siv Krüger Claussen, Erling Andersen, Anne Froholdt, Sigrun Randen, Hilde Presberg, Monica Wigemyr, Linda Margareth Pedersen, Bendik Slagsvold Winsvold, Mads Peder Rolfsen, Christian Helllum, Karianne Wiger Gammelsrud, Maria Dehli Vigeland, Benedicte Alexandra Lie, Siri Tennebø Flåm, Magnus Dehli Vigeland, Marianne Thorsø, Knut Morten Huneide, Veronica Sørensen, Olav Lutro, Thor Einar Holmgard

**Affiliations:** 1 Research and Communication Unit for Musculoskeletal Health(FORMI), Oslo University Hospital HF, Ullevål, Postbox 4956, Nydalen, 0424 Oslo, Norway; 2 Department of Physical Medicine and Rehabilitation, Oslo University Hospital HF, Ullevål, Postbox 4956, Nydalen, 0424 Oslo, Norway; 3 Department of Radiology, Haukeland University Hospital, Jonas Liesvei 65, 5021 Bergen, Norway; 4 Department of Rheumatology, Østfold Hospital Trust, PB 300, 1714 Grålum, Norway; 5 Department of Physical Medicine and Rehabilitation, St. Olavs Hospital, Trondheim University Hospital, Postbox 3250, Torgarden, NO-7006 Trondheim, Norway; 6 Oslo Centre of Biostatistics and Epidemiology, Research Support Services, Oslo University Hospital, Postbox 4950, Nydalen, 0424 Oslo, Norway; 7 Department of Physical Medicine and Rehabilitation, Haukeland University Hospital, Helse Bergen HF, Box 1, 5021 Bergen, Norway; 8 Department of Physiotherapy, Oslo Metropolitan University, PO box 4 St. Olavs plass, NO-0130 Oslo, Norway

**Keywords:** Modic changes, Low back pain, Clinical characteristics, Diagnostic accuracy, Short tau inversion recovery, Bone marrow edema, Magnetic resonance image, Back pain intensity, Springing test

## Abstract

**Background:**

Modic Changes (MCs, magnetic resonance imaging (MRI) signal changes in the vertebral bone marrow extending from the vertebral endplate) may represent a subgroup of nonspecific chronic low back pain that could benefit from a specific management. The primary aim was to compare clinical characteristics between patients with type 1 versus type 2 MCs. The secondary aim was to explore associations between clinical characteristics and MC related short tau inversion recovery (STIR) signals.

**Methods:**

This cross-sectional study used baseline data prospectively collected between 2015 and 2017 on the 180 patients included in the AIM-study (Antibiotics In Modic changes), a randomized controlled trial in a Norwegian hospital out-patient setting of patients with chronic low back pain, a lumbar disc herniation within the last 2 years, low back pain intensity score ≥ 5 (on a 0–10 scale) and current type 1 or type 2 MCs at the previously herniated lumbar disc level. We used prespecified clinical characteristics including self-report measures, physiologic measures and functional measures from clinical history and examination. The diagnostic accuracy of various clinical characteristics to discriminate between patients with type 1 MCs (with or without additional type 2 MCs) and patents with type 2 MCs only (not type 1) were assessed by calculating the area under the receiver-operating curve. We assessed the correlations of clinical characteristics with details of MC related STIR signal increase.

**Results:**

No clinical characteristic differed between patients with type 1 (*n* = 118) versus type 2 (but not type 1) (*n* = 62) MCs. The clinical characteristics showed no/minor differences or no/weak correlations with MC related STIR signal increase. Patients with a positive Springing test (at any lumbar level) had slightly less volume of STIR signal increase than those with a negative test (mean difference 1.3 on a 0–48 scale, 95% CI 0.3 to 2.3).

**Conclusion:**

Clinical characteristics were similar for patients with type 1 MCs and patients with type 2 MCs, and showed no clinically relevant correlations with MC related STIR signal increase.

**Trial registration:**

ClinicalTrials.gov NCT02323412, First registered 23 December 2014

## Background

Low back pain (LBP) is a leading global cause of disability [1] and a large majority of patients have nonspecific back pain without a clear pathoanatomical diagnosis [2]. It has been proposed that patients with Modic Changes (MCs) may represent a subgroup of nonspecific chronic LBP that could benefit from a specific management [3–6]. MCs are magnetic resonance imaging (MRI) signal changes in the vertebral bone marrow extending from the vertebral endplate. An association between LBP and MCs has been supported in systematic reviews [7, 8], but the association is inconsistent [9].

MCs have been proposed to be associated with a specific clinical profile [4, 10, 11]. In a cross-sectional study of a population based sample of 40-years old Danes, the clinical profile differed between people with MCs and disc degeneration compared to those with disc degeneration alone [4]. Of the 23 variables tested, the groups differed in duration and severity of LBP, previous disc herniation, heavy physical workload, heavy smoking, reduced physical activity at work and in leisure time, sick leave, pain on movement, inability to activate lumbar multifidus muscles and lumbar pain tolerance. A study of Chinese individuals found a similar picture, but only for patients with MCs in the lower lumbar spine [12].

MCs are defined into three types on MRI [13] that are considered to represent different stages of the same histopathological process [14]. Type 1 (edema type) is hypo-intense on T1- and hyper-intense on T2-weighted MRI, type 2 (fatty type) is hyper-intense on T1- and iso- or hyper-intense on T2, and type 3 (sclerotic type) is hypo-intense on T1- and T2. These definitions apply to non-fat saturated T2 images only. Individual MCs can change type over time [15]. Histopathological, inflammatory, biochemical and genetic studies support the differentiation between MC types. There is inflammation of the bone marrow, possibly more in type 1 than type 2 MCs [16, 17]. Ohtori et al. found more TNF-immunoreactive cells in endplates adjacent to type 1 compared to type 2 MCs [18] and Rannou et al. found higher CRP values in type 1 compared to both type 2 or no MCs [11].

Clinical features of the different MC types are poorly studied. Subjects with type 1 MCs have reported more intense pain than patients with type 2 MCs, and conversion from type 1 to type 2 has been associated with decreased pain [6, 11, 19–23]. Knowledge about clinical features is important, forming the primary basis for further management. For instance, patients with type 1 MCs may respond differently to treatment compared to patients with type 2 MCs [24–26]. Knowledge about potential important clinical differences between MC types can potentially reduce unnecessary treatment and imaging. Differences in clinical profiles between type 1 and type 2 MCs would also suggest that these two imaging phenotypes deserve to be viewed as separate entities. Hence, it is important to look at clinical differences other than just pain intensity. Finally, elucidating a clinical profile for each MC type could point to its etiology.

Biopsies of MCs have indicated that inflammatory changes, which could affect clinical features, may be present in both type 1 and type 2 MCs [16]. Findings on fat suppression MRI series have indicated that edema may also be present in type 2 (fatty type) MCs [27]. Thus, it is relevant to assess clinical features in relation to inflammatory edema across both MC types. Short tau inversion recovery (STIR) series are sensitive to edema and can show signal increase that reflect symptomatic or asymptomatic edema-like bone marrow lesions.

The primary aim of the present study was to compare clinical characteristics between patients with type 1 versus type 2 MCs. The secondary aim was to explore associations between clinical characteristics and MC related STIR signals.

## Methods

The present study is a cross-sectional study based on baseline data from a randomized controlled trial comparing amoxicillin to placebo in patients with chronic LBP and MCs (the Antibiotics In Modic changes (AIM)-study) [28]. The AIM-study was approved by the Regional Committees for Medical Research Ethics in south east Norway (REK Sør-Øst), was registered at ClinicalTrials.gov by December 2014 under the identifier: NCT02323412, and monitored by the Clinical Trial Unit, Oslo University Hospital. The trial was performed and reported in accordance with the Helsinki declaration. All patients gave written, informed consent to participate in the trial. Funding was granted by a governmental organisation (Helse Sør-Øst and Helse Vest), which did not have any role in planning, performing or reporting of the trial. A patient representative was a member of the Scientific Board of the study, which effected all the major decision from planning and design of the study, to the dissemination of the study results. The patient representative assessed the burden of the time and efforts required to participate in the trial.

### Eligibility criteria and study population

Participants with chronic LBP from all health regions in Norway were recruited at six participating hospitals’ outpatient clinics between June 2015 and September 2017.

Adults 18 to 65 years of age who presented with LBP of more than 6 months duration and type 1 and/or type 2 MCs were eligible for participation in the trial. Patients had to have a pain intensity score of ≥5 on a 0–10 Numerical Rating Scale (NRS) (mean of three NRS scores; current LBP, the worst LBP within the last 2 weeks, and usual/mean LBP within the last 2 weeks). MCs had to be confirmed on a study-specific MRI, have height ≥ 10% of vertebral height and diameter > 5 mm, and be present at a level with previous lumbar disc herniation verified on MRI within the preceding 2 years.

Patients were excluded if they had any specific diagnosis that could explain the low back symptoms (e.g. tumor, fracture, spondyloarthritis, infection, spinal stenosis), former low back surgery (L1 – S1) other than for disc herniation (e.g. fusion, decompression, disc prosthesis), or former surgery for disc herniation < 12 months before inclusion. Further exclusion criteria were use of opioids except codeine/tramadol, and patient unlikely to complete the AIM-study. See the trial protocol for a complete list of eligibility criteria (available at ClinicalTrials.gov).

### MRI

The study-specific MRI used in this investigation was performed at median 22 (interquartile range 15–29) days before the baseline measurements, and included standard T1- and T2 weighted fast spin echo images and sagittal STIR images. The same MRI protocol and 1.5 T scanner type (Magnetom Avanto with B19 software, Siemens) were used at all study sites. An integrated spine array coil was applied, but no surface coils. Imaging parameters for the sagittal images used in the present study were as follows: T1: repetition time 575 ms, echo time 11 ms, matrix 384 × 269, echo train length 5; T2: repetition time 3700 ms, echo time 87 ms, matrix 384 × 269, echo train length 17; STIR: repetition time 5530 ms, echo time 70 ms, inversion time 160 ms, matrix 320 × 224, echo train length 20. Field of view was 300 mm × 300 mm and slice thickness / spacing was 4.0 mm / 0.4 mm for all three sequences.

All sagittal slices were used to grade T1/T2- and STIR findings. MC types were defined by T1/T2 characteristics alone; type 1 as clearly hypo-intense on T1 and hyper-intense on T2, type 2 as hyper-intense on T1 and iso- or hyper-intense on T2. Patients were allocated to the type 1 MC group if their current study MRI showed MCs of primary (most extensive) or secondary type 1 at a level (superior or inferior endplate) with prior disc herniation verified on MRI within the last 2 years. Patients were allocated to the type 2 MC group if their study MRI showed MCs of primary or secondary type 2 – but not primary or secondary type 1 – at a level with MRI-verified disc herniation within the last 2 years. Patients with both type 1 MCs and type 2 MCs (at previously herniated disc levels) were hence allocated to the type 1 MC group. Thus, we were able to compare patients with type 1 MCs (and possibly type 2) versus type 2 MCs only (and not type 1).

As this was required for inclusion, all MCs used for MC group classification had height ≥ 10% of vertebral height and diameter > 5 mm on T1−/T2-weighted fast spin echo images. No criteria were predefined for which minimum size STIR signal changes should have to be reported.

We assessed MC related STIR signal increase (compared to normal vertebral body marrow) at 12 endplates (Th12-S1) using the following variables defined and selected prior to analysis:

I. Volume of STIR signal increase

Each of the 12 endplates was given a STIR volume score based on the volume of STIR signal increase in percent out of the total vertebral body volume (0 = no STIR signal, 1 = < 10%, 2 = < 25%, 3 = 25–50%, 4= > 50%). The STIR signal volume was visually estimated by taking into account the affected area on all images. A total sum score (possible values 0–48) for the 12 endplates was then calculated by summing up the score values for each individual endplate.

II. Maximum STIR signal intensity

The maximum intensity of MC related STIR signal increase at any endplate, recalculated and reported as a percentage on a STIR signal intensity scale ranging from normal vertebral body intensity (0%) to cerebrospinal fluid intensity (100%) (possible values 0–100). If maximum STIR signal intensity was reported for more than one endplate, the highest value was used.

III. Number of endplates with STIR signal increase

The number of endplates with MC related STIR signal increase (possible values 0–12).

Two radiologists independently classified patients into the type 1 or the type 2 MC group (kappa = 0.65, good inter-observer agreement [29]), and solved all disagreements on MC type group by discussion. Both radiologists independently evaluated the presence of MC related STIR signal increase (kappa ≥0.83, very good agreement), its volume, and its intensity relative to normal bone marrow and cerebrospinal fluid. If they disagreed on presence or volume of STIR signal increase, a third radiologist evaluated the STIR images, and the majority rating was used. For intensity measurements, we used the mean of two radiologists’ values. All radiologists had more than 10 years’ experience in musculoskeletal MRI.

### Clinical information and outcomes

All clinical information from history and examination was collected and reported by trial care givers (medical doctors or physiotherapists), who had available patients’ MC type group, but not their STIR findings. The patient-reported outcome measurements included the Roland and Morris Disability Questionnaire (RMDQ), LBP and leg pain intensity, Oswestry Disability Index (ODI) and the health-related quality of life (the EQ-5D). Background characteristics included age, gender, body mass index (BMI), ethnicity, marital status, educational level, work status, physical work load, leisure time activity, smoking habits, subjective health complaints (SHC) [30], emotional distress (Hopkins Symptom Checklist–25 HSCL-25) [31], fear-avoidance beliefs (FABQ work/physical activity) [32], LBP history/duration (including former treatment), comorbidities and pain medications. A detailed description of all outcomes collected in the trial is found in the trial protocol (available at ClinicalTrials.gov).

Table 1 describes the clinical characteristics of interest, pre-specified before data were available, with a rationale behind why they were chosen.

**Table 1:** Clinical characteristics of interest with rationale

Description	Rationale
Clinical History/Questionnaires	
**LBP intensity** [33] Mean of three 0–10 Numeric Rating Scales: current LBP, the worst LBP within the last 2 weeks, and usual/mean LBP within the last 2 weeks	Type 1 MCs are reported to be more strongly related to back pain than type 2 MCs [6, 11, 19–23, 34]
**Leg pain intensity** [35] 0–10 Numeric Rating Scales, last week.	Type 1 MCs may imply a slower initial decrease in sensory pain, but not leg pain intensity, compared to type 2 MCs in patients with radiculopathy [36]
**Duration of back pain** Time since onset of present back pain	Type 1 MCs reflect an active process and are commonly considered to develop before type 2 MCs, which may reflect a chronic process [13]
**Frequency of LBP** Number of days last 4 weeks with LBP and number of hours per day (average of 4 weeks) with back pain	Measures of frequency of LBP are found to be higher in patients with MCs than in patients without MCs [4]
**Pain on movement** Effect of walking on pain and effect of physical exercise on pain (Q: “What effect does the following activities have on your present pain”?, alternative responses for both walking and physical exercise were “worse”, “same”, “improved”, “unsure” or “not applicable”).	Pain on movement at physical examination was one of the most strongly significant discriminators between patients with and without MCs [4]
**LBP variation** Constant or intermittent LBP (Q: “Is the pain constant or intermittent throughout the day”?, alternative responses were “constant pain” or “intermittent pain”)	Constant pain is a clinical sign associated with regular spondylodiscitis [37], and low-grade disc infection is a hypothesized cause of MCs [38]
**Previous operation for disc herniation** If the patient had been operated for disc herniation, MCs had to found at an operated level for the patient to be included in the study	Following lumbar discectomy, type 2 could be more common than type 1 MCs at the operated level [39]. There is a trend toward less improvement of LBP post-discectomy with type 1 compared to type 2 or no MCs [40].
**Sleep disturbance** Assessed by Oswestry Disability Index- item 7, alternative responses were “my sleep is never disturbed by pain”, “my sleep is occasionally disturbed by pain”, “because of pain I have less than 6 h sleep”, “because of pain I have less than 4 h sleep”, “because of pain I have less than 2 h sleep” and “pain prevents me from sleeping at all”	Night pain was more common in type 1 MCs when compared to no MCs [41] or to type 2 MCs [11], and night pain is a clinical sign associated with regular spondylodiscitis [37] (low-grade disc infection is a hypothesized cause of MCs).
**LBP prevents sitting** Assessed by Oswestry Disability Index- item 5, alternative responses were: “I can sit in any chair as long as I like”, “I can only sit in my favorite chair as long as I like”, “pain prevents me sitting more than one hour”, “pain prevents me from sitting more than 30 min”, “pain prevents me from sitting more than 10 min”, and “pain prevents me from sitting at all”)	Explorative outcome.
Physical Examination	
**Aggravation of pain by flexion of lumbar spine** Recorded “pain” or “no pain” during lumbar spine flexion	Pain on lumbar movement (flexion, extension or lateral flexion) may discriminate between patients with and without MCs [4]
**Aggravation of pain by extension of lumbar spine** Recorded “pain” or “no pain” during lumbar spine extension	Pain on extension could be associated with MC type 1 [41, 42]
**Springing test for pain** In our study, Springing test was positive if the patient reported pain with pressure applied to lumbar transverse processes. In these analyses, we defined the Springing test as positive if it was positive anywhere in the lumbar spine.	Potential discriminator between patients with and without MCs [4]. Spinal tenderness is associated with spondylodiscitis (low-grade disc infection is a hypothesized cause of MCs) [37].

*LBP* Low back pain, *MCs* Modic Changes

### Statistical analysis

Differences in baseline characteristics between patients with type 1 versus type 2 MCs were analyzed by student’s t-test for normally distributed variables, by Mann-Whitney U test for non-normally distributed variables and by Chi-squared test for categorical data.

The diagnostic accuracy of each clinical variable to distinguish between type 1 and type 2 MCs was analyzed by calculating the area under the receiver operating characteristic curve (AUC) with its 95% CI (the term ‘diagnostic accuracy’ does not imply that MC type 1 s a diagnosis). The AUC represents the probability that a randomly chosen individual with type 1 MCs is (correctly) rated or ranked with greater suspicion than a randomly chosen individual with type 2 MCs, and can be interpreted as the clinical characteristic’s ability to discriminate between the two MC types [43]. Statistical analyses were performed with type 1 MCs defined as abnormal index test. An AUC > 0.5 was interpreted as an ability of the clinical characteristic to favor those with type 1 MCs, while AUC < 0.5 was interpreted as an ability of the clinical characteristic to favor those with type 2 MCs. For dichotomized variables we calculate sensitivity, specificity, positive likelihood ratio (PLR) and negative likelihood ratio (NLR). PLR and NLR were used instead of positive predictive value and negative predictive value, as the latter two require a valid estimate of prevalence of MC type 1 in relation to MC type 2 [44].

We analyzed 118 cases (type 1 MCs) and 62 controls (type 2 MCs), which meant we were able to detect (α = 0.05, β = 0.1) AUC larger than 0.628 (using http://www.biosoft.hacettepe.edu.tr/easyROC/). AUC values < 0.6 have been regarded as uninformative, and values 0.6 to 0.7 as indicating poor discrimination [34]. We thus regarded our sample size to be sufficiently large.

We used Pearson r, or Spearman rho, to analyze the associations between continuous clinical variables and STIR findings. We regarded correlation coefficient values < 0.10 as negligible correlation, values 0.10–0.39 as weak, values 0.40–0.69 as moderate, values 0.70–0.89 as strong and values > 0.90 as very strong correlation [45]. Categorical clinical variables were dichotomized by using Liu’s method for estimating the cutoff point on ROC curves for MC types. Dichotomous variables were analyzed by a t-test for associations with volume and intensity of STIR signal increase, and by Mann-Whitney U-test for associations with number of endplates with STIR signal increase.

We did not correct for multiple testing, as it was not the individual clinical characteristic’s association with the MC type that was of interest, but rather an exploration of various clinical characteristics together. Occasional false positive associations were therefore of less concern. Also, we did have an a priori justification for checking each clinical characteristic.

All analyses were performed using software package Stata version 15.

## Results

**Fig. 1:**
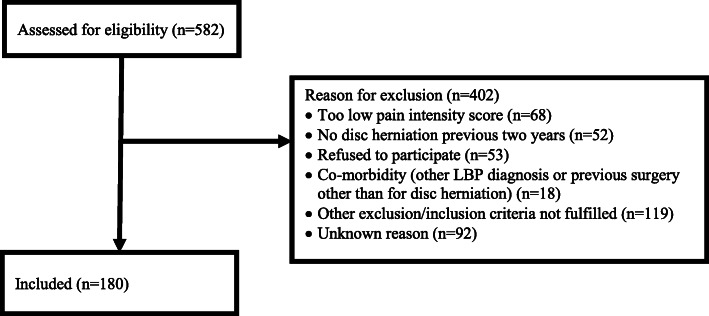
Flowchart. LBP Low back pain

We included 180 patients to the trial and in this study, 118 in the type 1 and 62 in the type 2 MC group. Reasons for exclusion into the trial are described in Fig. 1. Background characteristics are described in Table 2. Patients with type 1 MCs had somewhat lower BMI (mean difference: -1.3, 95% CI − 2.5 to 0.0) and were less likely to take opioids than patients with type 2 MCs (24% versus 44%, *p* = 0.006). There were no other differences in the background characteristics between patients with type 1 and type 2 MCs. No clinical characteristic of interest had more than three (1.7%) missing observations.

**Table 2:** Background characteristics

	N	Type 1 MCs	Type 2 MCs	*P*-value
Age, mean ± SD	180	45.3 ± 9.2	44.4 ± 8.6	0.54
Female, n (%)	180	70 (59%)	35 (56%)	0.71
Body mass index (BMI), mean ± SD	178	25.6 ± 4.0	26.9 ± 4.1	0.046
Smoking, n (%)	178	31 (27%)	15 (24%)	0.71
Educational level, n (%)	177			0.41
Primary school (9 years)		12 (10%)	7 (11%)	
High school (12 years)		48 (42%)	30 (48%)	
College/University < 4 year		28 (24%)	17 (27%)	
College/University ≥4 year		27 (23%)	8 (13%)	
Comorbidities, n (%)				
Diabetes	180	2 (2%)	1 (2%)	0.97
Psychiatric disease	180	5 (4%)	3 (5%)	0.85
Obesity	180	19 (16%)	10 (16%)	0.98
RMDQ, mean ± SD	178	12.6 ± 4.0	13.0 ± 4.6	0.58
Low back pain intensity, 0–10 NRS, mean ± SD	177	6.4 ± 1.2	6.2 ± 1.6	0.44
Leg pain intensity, 0–10 NRS, mean ± SD	179	3.0 ± 2.6	3.6 ± 2.5	0.17
EQ-5D, median (IQR)	179	0.60 (0.46–0.68)	0.56 (0.36–0.67)	0.28^a^
Emotional distress (HSCL −25) ≥1.75, n (%)	179	26 (22%)	21 (34%)	0.09
FABQ physical activity, 0–42, mean ± SD	179	12.2 ± 6.0	11.4 ± 5.7	0.39
FABQ work, 0–42, mean ± SD	176	17.0 ± 11.8	19.8 ± 11.9	0.15
Duration of low back pain, years median (IQR)	177	3 (1.7–6.3)	3.3 (1.3–6)	0.47^a^
Physical workload, n (%)	151			0.14
Job requires walking and lifting a lot or physically heavy work		28 (28%)	20 (39%)	
Level with Modic change, n (%)				
L1/L2	180	0	0	–
L2/L3	180	4 (3%)	0	0.14
L3/L4	180	7 (6%)	5 (8%)	0.59
L4/L5	180	52 (44%)	25 (40%)	0.63
L5/S1	180	84 (71%)	48 (77%)	0.37
Concomitant medication, n (%)				
Analgesics, any ^b^		77 (65%)	46 (74%)	0.22
Opioids (tramadol or codeine)		28 (24%)	27 (44%)	0.006

RMDQ Roland-Morris Disability Questionnaire. Pain and disability measure, ranges from 0 to 24, with a lower score indicating less severe pain and disability [46, 47]

NRS Numerical Rating Scale. A mean of three NRS scores; current pain, worst pain within the last 2 weeks, and usual/mean pain within the last 2 weeks. Used for low back pain intensity [33] and leg pain intensity [35]

EQ-5D Health related quality of life scores (EuroQoL -5D5L, version 2.0) [61]. Measured on 5 dimensions: mobility, self-care, usual activities, pain/discomfort and anxiety/depression, with a score 1–5 on each dimension. These values are converted to a single summary index by applying a ‘crosswalk value set’ for UK, giving a score from −0.59 to 1.0 (higher scores indicate a higher quality of life)

HSCL Hopkins Symptom Checklist–25 [31]. A measure of emotional distress

FABQ Fear-avoidance beliefs Questionnaire [32]

IQR Interquartile range (25th percentile - 75th percentile)

^a^Non-parametric test

^b^Including paracetamol/acetaminophen, NSAIDs, phenazone, acetylsalicylic acid and opioids (tramadol or codeine)

### Clinical characteristics and MC types

The distribution of each clinical characteristic by type 1 and type 2 MCs is summarized in Table S1 and Figure S1 in the Supplementary Appendix.

We found no statistically significant difference in any clinical characteristic between the two MC types (Table 3). The clinical characteristic variables showed poor ability to distinguish between type 1 and type 2 MCs with AUCs ranging from 0.42 to 0.55. Estimates of the diagnostic accuracy of all the clinical characteristics for MC types are summarized in Table 3.

**Table 3:** Diagnostic accuracy of various clinical characteristics to separate type 1 from type 2 Modic changes

	N	AUC (95% confidence interval)
Low back pain intensity (0–10 NRS score)	178	0.53 (0.44–0.63)
Leg pain intensity (0–10 NRS score)	179	0.44 (0.35–0.52)
Duration of back pain	179	0.53 (0.44–0.63)
Number of days last 4 weeks with low back pain	177	0.51 (0.47–0.56)
Number of hours per day (mean of last 4 weeks) with back pain	177	0.48 (0.39–0.56)
Pain worse when walking	178	0.50 (0.43–0.58)
Pain worse when exercising	177	0.55 (0.47–0.63)
Constant pain	178	0.54 (0.48–0.61)
Previous operation for disc herniation	180	0.54 (0.47–0.60)
Sleep disturbance (ODI sleep item score)	177	0.42 (0.33–0.50)
Back pain prevents sitting (ODI sitting item score)	178	0.43 (0.35–0.51)
Aggravation of pain by flexion of lumbar spine	177	0.52 (0.45–0.59)
Aggravation of pain by extension of lumbar spine	177	0.53 (0.45–0.60)
Springing test positive	180	0.52 (0.46–0.57)

*AUC* Area under the receiver operating characteristic curve, *NRS* Numerical rating score, *ODI* Oswestry Disability Index

### Clinical characteristics and STIR findings

There were only negligible or weak correlations between the clinical characteristics and the STIR variables (Tables S3, S4, S5 in the Supplementary Appendix). Total volume of MC related STIR signal increase was weakly correlated to age (r 0.18) and low BMI (r − 0.14) (Tables S3a) and was mean 1.3 points lower on the 0–48 point scale for patients with versus patients without a positive Springing test (Table S3b). Maximum STIR signal intensity was weakly correlated to the number of days with back pain last 4 weeks (r 0.19) and duration of back pain (r − 0.13) (Table S4a). The number of endplates with STIR signal increase was weakly correlated to age (rho 0.17) (Table S5a) and was smaller in patients who had versus patients who had not been disc operated (p 0.040, median 2 in both groups) (Table S5b).

## Discussion

This cross-sectional study found no differences in predefined clinical characteristics between patients with type 1 and patients with type 2 MCs. All correlations between MC related STIR signals and clinical characteristics were weak or negligible. We did observe some statistically significant associations and small differences, but due to the large number of tests performed there were no more than what we could expect by chance. These results suggest that one cannot distinguish patients with type 1 MCs from patients with type 2 MCs only based on clinical symptoms and signs. Our findings are in accordance with a study on Dutch military personnel which reported no differences in various clinical tests between type 1 and 2 MCs [48] and a study which did not report any difference in duration of symptoms between type 1 and type 2 MCs in hospitalized patients [11].

The slightly higher BMI in the type 2 versus the type 1 MC group may be due to multiple testing and is unlikely to be clinically relevant. A previous study reported that type 2 MCs were associated with fat mass and suggested a metabolic mechanism behind the fatty marrow in type 2 MCs [49]. However, type 2 MCs were not related to weight in a study of LBP patients [50].

Our observation that volume of and number of endplates with MC related STIR signal tended to increase with higher age might suggest an underlying degenerative process. Indeed, MCs are linked to disc degeneration in both cross-sectional and longitudinal studies [4, 51, 52]. However, our observed correlations were weak and do not exclude other non-degenerative mechanisms behind MCs.

Our finding that patients with a positive Springing test had slightly less volume of STIR signal increase was opposite of what we expected and makes little biological sense [4]. It is difficult to explain and may be due to chance.

As we did not find sufficient candidates of clinical characteristics, and to avoid further problems with multiple testing, we did not perform a multivariate regression analysis.

To our knowledge, no previous study has investigated the association between clinical characteristics and MC related STIR signal increases in patients with nonspecific chronic LBP. It has been shown that vertebral bone marrow edema on gadolinium-enhanced MRI is related to LBP and lumbar tender points in elderly patients with degenerative scoliosis [53] and similarly, bone marrow edema on MRI related to pain in knees and ankles [54, 55].

The main limitation of our study is that it was based on a sample that was not representative of LBP patients in general, but included patients with particularly strong symptoms and already verified MCs at the level of a previous disc herniation. The fact that we were able to enroll many more patients with type 1 MCs than type 2 MCs supports the possibility of selection bias. In addition, restricting analysis to a subgroup with a limited range of pain intensity is likely to lower correlation coefficients for variables related to pain intensity [56]. Further, as we did not include patients without LBP, we cannot conclude about an association between presence of LBP and type of MCs or degree of MC related STIR signal increase.

Another limitation is that the type 1 MC group also included patients with both type 1 and type 2 MCs. This might have obscured any differences in clinical characteristics between a more pure type 1 MC group and a pure type 2 MC group. Further, we based the MC type group on MCs found at a level with a previous disc herniation, since such MCs were target for the treatment tested in our trial, and some patients had other types of MCs at other levels. However, as many patients have a mix of MC types, comparing clinical features between pure MC type groups would be less clinically relevant.

The assessors that performed the physical examinations had a heterogeneous clinical experience that could reduce the reliability of the examinations, and thus increase the risk of overlooking their true relations to other variables [57]. The reliability was acceptable for the Springing test [58], but poor or modest for many physical tests, in previous studies [59]. Accordingly, more clinical experience of the clinicians may not improve reliability [59].

Strengths of our study include strictly standardized MRI technique and MRI evaluation by multiple experienced radiologists, which can improve the reliability of MRI reports [60]. Further, data collection was systematic and prospective, and the data were almost complete. Despite the study had limitations, we would expect it to have revealed at least some relevant associations if strong true associations actually existed in the chronic LBP population.

## Conclusion

Our study of selected patients with chronic LBP showed no differences in clinical features between patients with type 1 MCs and patients with type 2 MCs. There were only weak associations between clinical patient characteristics and MC related STIR signal increase.

## Supplementary information

**Additional file 1: Table S1.** Distribution of categorical clinical characteristic within each Modic change type. **Figure S1.** Distribution of continuous clinical characteristics within each Modic change type. **Table S2.** Diagnostic accuracy of various clinical characteristics to separate type 1 from type 2 Modic changes. **Table S3**a. Volume of MC related STIR signal increase vs continuous clinical variables. **Table S3**b. Volume of Modic change related STIR signal increase vs dichotomous clinical variables. **Table S4**a. Maximum intensity of Modic change related STIR signal vs continuous clinical variables. **Table S4**b. Maximum intensity of Modic change related STIR signal vs dichotomous clinical variables. **Table S5**a. Number of endplates with Modic change related STIR signal increase vs continuous clinical variables. **Table S5**b. Number of endplates with Modic change related STIR signal increase vs dichotomous clinical variables.

## Data Availability

Requests to access data should be addressed to kjersti.storheim@medisin.uio.no. De-identified individual participant data (including data dictionary) will be available to medical researchers by request in accordance with local registration and ethical approval, when the article has been published until 1st of July, 2029. All proposals requesting data access will need to specify an analysis plan and will need approval of the scientific board before any data can be released.

## Abbreviations

MCs: Modic changes
MRI: Magnetic Resonance Image
STIR: Short tau inversion recovery
AIM: Antibiotics in Modic changes
LBP: Low back pain
TNF: Tumor Necrosis Factor
CRP: C-reactive protein
REK: Regionale komiteer for medisinsk og helsefaglig forskningsetikk (Regional Committees for Medical and Health research Ethics)
NRS: Numerical Rating Scale
RMDQ: Roland-Morris Disability Questionnaire
ODI: Oswestry Disability Index
EQ-5D: EuroQol − 5 dimensions (health-related quality of life score).
BMI: body mass index,
SHC: subjective health complaints
HSCL-25: Hopkins Symptom Checklist–25
FABQ: Fear-avoidance beliefs questionnaire
AUC: Area under the receiver operating characteristic curve
PLR: Positive likelihood ratio
NLR: Negative likelihood ratio
CI: Confidence interval
