# Supplementary material for: Association of Modic change types and their short tau inversion recovery signals with clinical characteristics- a cross sectional study of chronic low back pain patients in the AIM-study
Source: BMC Musculoskelet Disord. 2020 Jun 10;21:368. doi: 10.1186/s12891-020-03381-4 (PMC7285575; doi:10.1186/s12891-020-03381-4)
Supplement: Supplementary file 1 — Additional file 1: Table S1. Distribution of categorical clinical characteristic within each Modic change type. Figure S1. Distribution of continuous clinical characteristics within each Modic change type. Table S2. Diagnostic accuracy of various clinical characteristics to separate type 1 from type 2 Modic changes. Table S3a. Volume of MC related STIR signal increase vs continuous clinical variables. Table S3b. Volume of Modic change related STIR signal increase vs dichotomous clinical variables. Table S4a. Maximum intensity of Modic change related STIR signal vs continuous clinical variables. Table S4b. Maximum intensity of Modic change related STIR signal vs dichotomous clinical variables. Table S5a. Number of endplates with Modic change related STIR signal increase vs continuous clinical variables. Table S5b. Number of endplates with Modic change related STIR signal increase vs dichotomous clinical variables. [file 12891_2020_3381_MOESM1_ESM.docx]

Supplementary Appendix

Table of Contents

[Table S1: Distribution of categorical clinical characteristic within each Modic change type 2](#_Toc42171580)

[Figure S1- Distribution of continuous clinical characteristics within each Modic change type 3](#_Toc42171581)

[Table S2 – Diagnostic accuracy of various clinical characteristics to separate type 1 from type 2 Modic changes 4](#_Toc42171582)

[Table S3a- Volume of MC related STIR signal increase vs continuous clinical variables 5](#_Toc42171583)

[Table S3b - Volume of Modic change related STIR signal increase vs dichotomous clinical variables 6](#_Toc42171584)

[Table S4a – Maximum intensity of Modic change related STIR signal vs continuous clinical variables 7](#_Toc42171585)

[Table S4b – Maximum intensity of Modic change related STIR signal vs dichotomous clinical variables 7](#_Toc42171586)

[Table S5a – Number of endplates with Modic change related STIR signal increase vs continuous clinical variables 8](#_Toc42171587)

[Table S5b – Number of endplates with Modic change related STIR signal increase vs dichotomous clinical variables 8](#_Toc42171588)

## Table S1: Distribution of categorical clinical characteristic within each Modic change type

|  | N | Type 1 MCs  n(%) | Type 2 MCs |
| --- | --- | --- | --- |
| Effect of walking on pain | 178 |  |  |
| Worse |  | 42(36%) | 22(35%) |
| Missing |  | 2(2%) | 0 |
| Effect of exercise on pain | 177 |  |  |
| Worse |  | 50(42%) | 33(53%) |
| Missing |  | 3(3%) | 0 |
| Pain variation | 178 |  |  |
| Intermittent pain |  | 84(71%) | 49(79%) |
| Constant pain |  | 33(28%) | 12(19%) |
| Missing |  | 1(1%) | 1(2%) |
| Previous operation for disc herniation | 180 | 22(19%) | 16(26%) |
| Sleep disturbance (ODI-item) | 177 |  |  |
| My sleep is never disturbed by pain |  | 3(3%) | 1(2%) |
| My sleep is occasionally disturbed by pain |  | 60(51%) | 25(40%) |
| Because of pain I have less than 6 hours sleep |  | 45(38%) | 23(37%) |
| Because of pain I have less than 4 hours sleep |  | 7(6%) | 11(18%) |
| Because of pain I have less than 2 hours sleep |  | 1(1%) | 1(2%) |
| Pain prevents me from sleeping at all |  | 0(0%) | 0(0%) |
| Missing |  | 2(2%) | 1(2%) |
| Sitting (ODI-item) | 178 |  |  |
| I can sit in any chair as long as I like |  | 10(9%) | 1(2%) |
| I can only sit in my favorite chair as long as I like |  | 19(16%) | 7(11%) |
| Pain prevents me from sitting more than one hour |  | 58(50%) | 33(53%) |
| Pain prevents me from sitting more than 30 minutes |  | 24(20%) | 17(27%) |
| Pain prevents me from sitting more than 10 minutes |  | 6(5%) | 3(5%) |
| Pain prevents me from sitting at all |  | 0(0%) | 0(0%) |
| Missing |  | 1(1%) | 1(2%) |
| Flexion of lumbar spine | 177 |  |  |
| Pain aggravated on flexion |  | 87(74%) | 42(70%) |
| Missing |  | 1(1%) | 2(3%) |
| Extension of lumbar spine | 177 |  |  |
| Pain aggravated on extension |  | 84(72%) | 40(67%) |
| Missing |  | 1(1%) | 2(3%) |
| Springing test | 180 |  |  |
| Positive |  | 103(87%) | 52(84%) |

ODI Oswestry Disability Index

## Figure S1- Distribution of continuous clinical characteristics within each Modic change type

LBP Low back pain

MC Modic change

## Table S2 – Diagnostic accuracy of various clinical characteristics to separate type 1 from type 2 Modic changes

|  | N (%) | Sensitivity  (%) | Specificity  (%) | Pos likelihood ratio  (95% CI) | Neg likelihood ratio  (95% CI) | AUC  (95% CI) |
| --- | --- | --- | --- | --- | --- | --- |
| LBP intensity-NRS | 178 | - | - | - | - | 0.53  (0.44 - 0.63) |
| Leg pain intensity-NRS | 179 | - | - | - | - | 0.44  (0.35 - 0.52) |
| Duration of back pain | 179 | - | - | - | - | 0.53  (0.44 - 0.63) |
| Number of days last 4 weeks with LBP | 177 | - | - | - | - | 0.51  (0.47 - 0.56) |
| Number of hours per day (mean of last 4 weeks) with back pain | 177 | - | - | - | - | 0.48  (0.39 - 0.56) |
| Pain worse when walking | 178 | 36.2  (27.5 - 45.7) | 64.5  (51.3 - 76.3) | 1.0  (0.7 - 1.5) | 1.0  (0.8 - 1.2) | 0.50  (0.43 - 0.58) |
| Pain worse when exercising | 177 | 43.5  (34.6 - 53.0) | 46.8  (34.0 - 59.9) | 0.8  (0.60 - 1.1) | 1.21  (0.89 - 1.65) | 0.45  (0.37 - 0.53) |
| Constant pain | 178 | 28.2  (20.3 - 37.3) | 80.3  (68.2 - 89.4) | 1.4  (0.8 - 2.6) | 0.9  (0.8 - 1.1) | 0.54  (0.48 - 0.61) |
| Previous operation for disc herniation | 180 | 81.4  (73.1 - 87.9) | 25.8  (15.5 - 38.5) | 1.1  (0.9 - 1.3) | 0.7  (0.4 - 1.3) | 0.54  (0.47 - 0.60) |
| Sleep disturbance (ODI sleep item score) | 177 | - | - | - | - | 0.42  (0.33 - 0.50) |
| Back pain prevents sitting (ODI sitting item score) | 178 | - | - | - | - | 0.43  (0.35 - 0.51) |
| Aggravation of pain by flexion of lumbar spine | 177 | 74.4  (65.5 - 82.0) | 30.0  (18.9 - 43.2) | 1.1  (0.9 - 1.3) | 0.9  (0.5 - 1.4) | 0.52  (0.45 - 0.59) |
| Aggravation of pain by extension of lumbar spine | 177 | 71.8  (62.7 - 79.7) | 33.3  (21.7 - 46.7) | 1.1  (0.9 - 1.3) | 0.9  (0.5 - 1.3) | 0.53  (0.45 - 0.60) |
| Springing test positive | 180 | 87.3  (79.9 - 92.7) | 16.1  (8.0 - 27.7) | 1.0  (0.9 - 1.2) | 0.8  (0.4 - 1.7) | 0.52  (0.46 - 0.57) |

For non-dichotomous clinical characteristics with AUC not different from 0.5, we did not calculate a cutoff value (hence no sensitivity, specificity, positive likelihood ratio and negative likelihood ratio).

AUC Area under the receiver operating characteristic curve

CI Confidence interval

LBP Low back pain

NRS Numerical rating score

ODI Oswestry Disability Index

## Table S3a- Volume of MC related STIR signal increase vs continuous clinical variables

|  | N | Correlation coefficient | 95% Confidence interval |
| --- | --- | --- | --- |
| LBP intensity-NRS | 178 | 0.02 | -0.13 to 0.17 |
| Leg pain intensity-NRS | 179 | 0.04 | -0.11 to 0.19 |
| Duration of back pain* | 178 | 0.06 | -0.08 to 0.21 |
| Days with LBP last 4 weeks** | 177 | 0.01 | -0.14 to 0.16 |
| Hours with LBP on a typical day** | 177 | 0.02 | -0.13 to 0.17 |
| Age | 180 | 0.18 | 0.03 to 0.32 |
| Body mass index (BMI) | 179 | -0.14 | -0.29 to 0.00 |
| RMDQ | 178 | 0.01 | -0.13 to 0.16 |
| ODI | 177 | -0.03 | -0.18 to 0.11 |
| EQ5D | 180 | 0.06 | -0.09 to 0.20 |
| HSCL | 179 | -0.05 | -0.19 to 0.10 |
| FABQ physical activity | 179 | 0.02 | -0.13 to 0.17 |
| FABQ work | 176 | -0.08 | -0.22 to 0.07 |

LBP Low back pain

NRS Numerical rating score

ODI Oswestry Disability Index

STIR Short tau inversion recovery

RMDQ Roland-Morris Disability Questionnaire

NRS Numerical Rating Scale

EQ5D Health related quality of life scores (EuroQoL -5D5L, version 2.0)

HSCL Hopkins Symptom Checklist–25. A measure of emotional distress

FABQ Fear-avoidance beliefs Questionnaire

* analyzed log transformed version of variable

** analyzed with Spearman correlation

## Table S3b - Volume of Modic change related STIR signal increase vs dichotomous clinical variables

|  | N | Not present, mean | Present, mean | Difference between groups | 95% confidence interval | P-value |
| --- | --- | --- | --- | --- | --- | --- |
| Pain worse when walking | 180 | 3.81 | 4.00 | -0.19 | -0.92 to 0.57 | 0.61 |
| Pain worse when exercising | 180 | 3.79 | 3.98 | -0.18 | -0.86 to 0.57 | 0.61 |
| Constant pain | 178 | 3.99 | 3.58 | 0.41 | -0.40 to 1.23 | 0.32 |
| Previous operation for disc herniation | 180 | 4.04 | 3.26 | 0.78 | -0.08 to 1.64 | 0.07 |
| Sleep disturbance (ODI sleep item score ≥2) | 177 | 4.06 | 3.77 | 0.28 | -0.43 to 0.99 | 0.43 |
| Pain prevents sitting (ODI sitting item score ≥3) | 178 | 4.12 | 3.34 | 0.78 | -0.00 to 1.56 | 0.051 |
| Aggravation of pain by flexion of lumbar spine | 177 | 4 | 3.81 | 0.19 | -0.61 to 0.99 | 0.63 |
| Aggravation of pain by extension of lumbar spine | 177 | 3.74 | 3.91 | -0.18 | -0.95 to 0.60 | 0.66 |
| Springing test positive | 180 | 4.96 | 3.70 | 1.26 | 0.25 to 2.26 | 0.01 |

ODI Oswestry Disability Index

STIR Short tau inversion recovery

## Table S4a – Maximum intensity of Modic change related STIR signal vs continuous clinical variables

|  | N | Correlation | 95% Confidence interval |
| --- | --- | --- | --- |
| LBP intensity-NRS | 166 | 0.07 | -0.09 to 0.22 |
| Leg pain intensity-NRS | 167 | -0.10 | -0.25 to 0.05 |
| Duration of back pain* | 166 | -0.13 | -0.27 to 0.03 |
| Days with LBP last 4 weeks** | 165 | 0.19 | 0.04 to 0.33 |
| Hours with LBP on a typical day** | 165 | 0.02 | -0.13 to 0.17 |
| Age | 168 | -0.08 | -0.23 to 0.07 |
| Body mass index (BMI) | 167 | -0.08 | -0.23 to 0.07 |
| RMDQ | 167 | -0.03 | -0.18 to 0.13 |
| ODI | 166 | -0.12 | -0.27 to 0.03 |
| EQ5D | 168 | 0.02 | -0.13 to 0.17 |
| HSCL | 167 | -0.06 | -0.21 to 0.09 |
| FABQ physical activity | 167 | -0.05 | -0.20 to 0.10 |
| FABQ work | 165 | -0.08 | -0.23 to 0.07 |

LBP Low back pain

NRS Numerical rating score

ODI Oswestry Disability Index

STIR Short tau inversion recovery

RMDQ Roland-Morris Disability Questionnaire

NRS Numerical Rating Scale

EQ5D Health related quality of life scores (EuroQoL -5D5L, version 2.0)

HSCL Hopkins Symptom Checklist–25. A measure of emotional distress

FABQ Fear-avoidance beliefs Questionnaire

* analyzed log transformed version of variable

** analyzed with Spearman correlation

## Table S4b – Maximum intensity of Modic change related STIR signal vs dichotomous clinical variables

|  | N | Not present, mean | Present, mean | Difference between groups | 95% confidence interval | P-value |
| --- | --- | --- | --- | --- | --- | --- |
| Pain worse when walking | 166 | 37 | 36 | 0.7 | -4.1 to 4.8 | 0.77 |
| Pain worse when exercising | 165 | 37 | 37 | 0.3 | -4.4 to 4.3 | 0.91 |
| Constant pain | 166 | 37 | 37 | -0.2 | -5.1 to 4.7 | 0.93 |
| Previous operation for disc herniation | 168 | 37 | 35 | 2.1 | -3.2 to 7.5 | 0.43 |
| Sleep disturbance (ODI sleep item score ≥2) | 166 | 38 | 36 | 2.3 | -2.0 to 6.6 | 0.29 |
| Pain prevents sitting (ODI sitting item score ≥3) | 167 | 38 | 35 | 3.1 | -1.7 to 8.0 | 0.20 |
| Aggravation of pain by flexion of lumbar spine | 165 | 35 | 37 | -2.1 | -7.1 to 2.8 | 0.40 |
| Aggravation of pain by extension of lumbar spine | 165 | 35 | 37 | -2.3 | -7.0 to 2.5 | 0.34 |
| Springing test positive | 168 | 39 | 37 | 2.6 | -3.5 to 8.7 | 0.41 |

ODI Oswestry Disability Index

STIR Short tau inversion recovery

## Table S5a – Number of endplates with Modic change related STIR signal increase vs continuous clinical variables

|  | N | Correlation* | 95% Confidence interval |
| --- | --- | --- | --- |
| LBP intensity-NRS | 178 | -0.03 | -0.18 to 0.12 |
| Leg pain intensity-NRS | 179 | 0.05 | -0.10 to 0.20 |
| Duration of back pain | 179 | 0.07 | -0.08 to 0.21 |
| Days with LBP last 4 weeks | 177 | 0.05 | -0.10 to 0.20 |
| Hours with LBP on a typical day | 177 | 0.05 | -0.10 to 0.20 |
| Age | 180 | 0.17 | 0.02 to 0.31 |
| Body mass index (BMI) | 179 | -0.08 | -0.22 to 0.07 |
| RMDQ | 178 | -0.07 | -0.22 to 0.07 |
| ODI | 177 | -0.03 | -0.18 to 0.12 |
| EQ5D | 180 | 0.04 | -0.10 to 0.19 |
| HSCL | 179 | -0.08 | -0.22 to 0.07 |
| FABQ physical activity | 179 | -0.03 | -0.18 to 0.11 |
| FABQ work | 176 | -0.05 | -0.20 to 0.10 |

LBP Low back pain

NRS Numerical rating score

ODI Oswestry Disability Index

STIR Short tau inversion recovery

RMDQ Roland-Morris Disability Questionnaire

NRS Numerical Rating Scale

EQ5D Health related quality of life scores (EuroQoL -5D5L, version 2.0)

HSCL Hopkins Symptom Checklist–25. A measure of emotional distress

FABQ Fear-avoidance beliefs Questionnaire

*Spearman correlation

## Table S5b – Number of endplates with Modic change related STIR signal increase vs dichotomous clinical variables

|  | N | Not present, median (IQR) | Present, median (IQR) | P-value |
| --- | --- | --- | --- | --- |
| Pain worse when walking | 178 | 2 (2 to 2) | 2 (2 to 2) | 0.60 |
| Pain worse when exercising | 177 | 2 (2 to 2) | 2 (2 to 3) | 0.16 |
| Constant pain | 178 | 2 (2 to 2) | 2 (2 to 2) | 0.43 |
| Previous operation for disc herniation | 180 | 2 (2 to 2) | 2 (2 to 2) | 0.040 |
| Sleep disturbance (ODI sleep item score ≥2) | 177 | 2 (2 to 2) | 2 (2 to 3) | 0.73 |
| Pain prevents sitting (ODI sitting item score ≥3) | 178 | 2 (2 to 2) | 2 (2 to 2) | 0.26 |
| Aggravation of pain by flexion of lumbar spine | 177 | 2 (2 to 3) | 2 (2 to 2) | 0.44 |
| Aggravation of pain by extension of lumbar spine | 177 | 2 (2 to 2) | 2 (2 to 2) | 0.40 |
| Springing test positive | 180 | 2 (2 to 4) | 2 (2 to 2) | 0.0502 |

ODI Oswestry Disability Index

STIR Short tau inversion recovery
